# Experience of violence and adverse reproductive health outcomes, HIV risks among mobile female sex workers in India

**DOI:** 10.1186/1471-2458-11-357

**Published:** 2011-05-20

**Authors:** Suvakanta N Swain, Niranjan Saggurti, Madhusudana Battala, Ravi K Verma, Anrudh K Jain

**Affiliations:** 1 HIV and AIDS Program, Population Council, 142 Golf Links, New Delhi - 110003. India; 2 International Center for Research on Women, Defence Colony, New Delhi - 110003. India; 3 Distinguished Scholar, Population Council, One Dag Hammarskjold Plaza, New York, NY 10017. USA

## Abstract

**Background:**

Female sex workers (FSWs) are a population sub-group most affected by the HIV epidemic in India and elsewhere. Despite research and programmatic attention to FSWs, little is known regarding sex workers' reproductive health and HIV risk in relation to their experiences of violence. This paper therefore aims to understand the linkages between violence and the reproductive health and HIV risks among a group of mobile FSWs in India.

**Methods:**

Data are drawn from a cross-sectional behavioural survey conducted in 22 districts from four high HIV prevalence states (Andhra Pradesh, Karnataka, Maharashtra, Tamil Nadu) in India between September 2007 and July 2008. The survey sample included 5,498 FSWs who had moved to at least two different places for sex work in the past two years, and are classified as mobile FSWs in the current study. Analyses calculated the prevalence of past year experiences of violence; and adjusted logistic regression models examined the association between violence and reproductive health and HIV risks after controlling for background characteristics and program exposure.

**Results:**

Approximately one-third of the total mobile FSWs (30.5%, n = 1,676) reported experiencing violence at least once in the past year; 11% reported experiencing physical violence, and 19.5% reported experiencing sexual violence. Results indicate that FSWs who had experienced any violence (physical or sexual) were significantly more likely to be vulnerable to both reproductive health and HIV risks. For example, FSWs who experienced violence were more likely than those who did not experience violence to have experienced a higher number of pregnancies (adjusted odds ratio [OR] = 1.2, 95% confidence interval [CI] = 1.0-1.6), ever experienced pregnancy loss (adjusted OR = 1.4, 95% CI = 1.2-1.6), ever experienced forced termination of pregnancy (adjusted OR = 2.4, 95% CI = 2.0-2.7), experienced multiple forced termination of pregnancies (adjusted OR = 2.2, 95% CI = 1.7-2.8), and practice inconsistent condom use currently (adjusted OR = 1.97, 95% CI: 1.4-2.0). Among FSWs who experienced violence, those who experienced sexual violence were more likely than those who had experienced physical violence to report inconsistent condom use (adjusted OR = 1.8, 95% CI: 1.4-2.3), and experience STI symptoms (adjusted OR = 1.3, 95% CI: 1.1-1.7).

**Conclusion:**

The pervasiveness of violence and its association with reproductive health and HIV risk highlights that the abuse in general is an important determinant for reproductive health risks; and sexual violence is significantly associated with HIV risks among those who experienced violence. Existing community mobilization programs that have primarily focused on empowering FSWs should broaden their efforts to promote reproductive health in addition to the prevention of HIV among all FSWs, with particular emphasis on FSWs who experienced violence.

## Background

In the recent past, violence against women has been identified as a major risk factor for acquiring HIV infection around the world [1-8]. In Asian countries, such as India, Indonesia and Cambodia, which are low prevalence settings with a concentrated HIV epidemic, there has been increased programmatic focus on female sex workers (FSWs) [9], as they have significantly higher HIV/sexually transmitted infection (STI) prevalence rates than the general population [10].

As a marginalized population group due to sex work lacking social and moral approval [11], FSWs are highly vulnerable to various forms of violence, perpetrated by the police, clients, pimps, agents and fellow sex workers. A growing body of evidence has highlighted the magnitude of violence against the FSW population in Asia and Africa [10,12-14]. In numerous countries, the police regularly rape and beat sex workers and demand bribes to avoid arrest. In Bangladesh, the National HIV Surveillance (1999-2000) reported that 52-60% of street-based sex workers had been raped by men in uniform and 41-51% had been raped by local criminals. In India, 70% of sex workers in a survey reported being beaten by the police and more than 80% had been arrested without evidence [14].

The link between domestic violence and HIV risk has also been documented in numerous studies across the South Asian region [9,14-16]. In India too, mounting evidence highlights the relevance of violence in understanding the reproductive health and HIV risks of sex workers -- low condom use with clients, high prevalence of STIs [9-11,15]. For example, a study in Karnataka indicates that 26% of FSWs had been beaten or raped in the past year; those who reported violence were significantly less likely to report condom use with clients, and were more likely to be infected with gonorrhea [11]. These and similar data indicate the need for studies that explore other reproductive health consequences among FSWs experiencing violence and the forms of violence that may both be a marker for and directly facilitate HIV risk among FSWs in India and elsewhere. Indeed, the experience of violence is recognized to have an adverse impact on the reproductive health outcomes of women in the general population, including unintended pregnancy [17,18], poor pregnancy outcomes [19,20], and gynecological morbidity [21,22], which may also be true for women in sex work.

Outside of India, studies examining the role of coercion in sex work and its association with adverse sexual and reproductive health outcomes report that sexual coercion/violence is strongly linked to HIV infection [13,23-26], and violence in general is a factor for poor reproductive health outcomes among FSWs [26] . As the effect of violence victimization (that is, physical and sexual violence) could differ in relation to both reproductive health risk and HIV risk among FSWs, studies are needed that examine these issues among the FSW population. The present study of mobile FSWs therefore aims to: (1) understand the prevalence of violence in this group; (2) explore the prevalence of sexual violence among FSWs who experienced violence; (3) understand the linkage between violence and reproductive health and HIV risks among FSWs; and (4) examine the differential effect of types of violence on reproductive health risks and HIV risks in this group.

## Methods

### Design

Data used in this study are obtained from a cross-sectional behavioral survey conducted among FSWs from June 2007 to September 2008 as part of a study on "Migration/Mobility and Vulnerability to HIV Among Male Migrant Workers and FSWs in High HIV prevalence states in India" funded by Avahan, the India AIDS Initiative of the Bill and Melinda Gates Foundation (BMGF). The participants were recruited from 22 districts across four states in southern (Andhra Pradesh, Karnataka, Tamil Nadu) and western (Maharashtra) India, identified as high epidemic states by the Indian National AIDS Control Organisation. The four states together estimated to have contributed to 72% of the HIV burden in India according to the sentinel surveillance of 2005 [27].

The study districts were identified using mapping and enumeration data on FSWs in each state, conducted independently by the State AIDS Control Society and Avahan (unpublished data). Districts with more than 2,000 reported FSWs were chosen (n = 22 districts; 5 districts each in the states of Andhra Pradesh, Karnataka and Tamil Nadu; 7 districts in Maharashtra). Field research started with the identification of sites where sex is solicited in each selected district. Sex solicitation sites were areas with either lesser or more number of sex workers and have included brothel areas and open solicitation areas (also mentioned as 'non-brothel sites' in this paper) such as roads, highways, bus stations, railway stations and market areas. The list of solicitation sites was used to define and select site clusters. Clusters were formed by combining smaller areas or segmenting the larger areas such that each cluster included approximately 500 FSWs. Three such clusters from each district were randomly selected to obtain a minimum of 150 eligible participants per district. Eligibility criteria included FSWs aged 18 years or older who had moved to at least two places in the past two years for sex work.

In each selected cluster, study participants were recruited through a two-stage sampling procedure conducted in selected brothel-based and open solicitation sites. Maps drawn for each selected cluster were used to list all sex worker locations. For brothel sites, lanes/small areas were selected in the first stage while brothel houses in each lane/small area were selected in the second stage. All FSWs in the selected brothel houses were interviewed using a screening tool. In the case of non-brothel sites, sex worker solicitation sites were selected in the first stage; and the day and timing of visits were systematically selected in the second stage. All FSWs found in the selected non-brothel sites during the selected time and day were interviewed using a screening tool.

The sample size for the study was determined using an estimated proportion of 30% non-condom use, an assumed difference of 3% increase in the proportion with every unit increase in mobility, a confidence level (CI) of 95% and power of 80%. In order to achieve the desired eligible sample size, a larger number of FSWs were contacted and screened using the tool that determined eligibility for participation in the detailed survey.

Across the entire study, about 94% (or 9,475) of FSWs who were initially contacted (10,075) agreed to be administered the screening questionnaire. From the screened sample, 59% (n = 5,611) were found eligible according to the criteria described above. Of the total eligible FSWs (5,611), 113 were excluded: 15 were not interviewed because they were below age 18 years, 21 refused to participate, 51 withdrew while being interviewed due to the demands of their clients, and for additional 26 the data were missing on socio-economic variables. This resulted in a total analytical sample of 5,498 FSWs.

Ethical approval for the study was obtained from the institutional review boards (IRBs) of the Population Council and the University of Manitoba, Canada. Verbal consent was obtained from all respondents prior to participation at each stage. For ethical reasons, only FSWs who were at least 18 years of age were interviewed. Participants were not provided any compensation for their time in the study but were given information on local organizations that provide services for treatment of STIs and condoms.

Interviews were conducted by trained researchers with multilingual expertise. All the researchers had at least five years of experience and a Graduate or Masters' degree in sociology, anthropology and/or statistics. Participants were asked to respond to a 45-minute interviewer-administered survey in the local language. Instruments were developed in English, translated into four local languages and then reviewed by study investigators who were fluent in English and the local language. Discrepancies were resolved in consultation with the Principal Investigator from the Population Council.

All interviews were conducted in private or public locations depending on the preference of the respondent. Locations for street-based FSWs included street corners, gardens, parks, and areas outside cinema halls. Data were collected using handheld PDAs (Palmtop Digital Accessories) in the states of Maharashtra, Andhra Pradesh and Tamil Nadu; and through printed questionnaires in Karnataka. This is the first time that PDAs have been used for a survey of FSWs in India [28,29]. In order to facilitate the acceptance of PDAs, respondents were told about the interviewing technique and shown how the PDA works. A customized PDA program was used to ensure the confidentiality of the large-scale sensitive data collected in the field and to reduce errors in data entry using the PDA. Data quality control and management for questionnaires involved immediate review by field staff after interviews to ensure accuracy and completion, same-day review by the field supervisor and weekly transport of survey forms to the data management team. Trained data entry officers then entered the survey data weekly and processed it monthly to verify consistency and accuracy, using SPSS software (Version 16.0). The consistency and quality of the data collected through the use of PDAs was assessed weekly using SPSS.

### Measures

Demographics were assessed via single items regarding age, education, marital status, duration of sex work, type of sex work, exposure to HIV prevention program and the degree of mobility of FSWs. The degree of mobility was defined on the basis of the responses to the question on the number of places the respondent had moved for sex work in the past two years: moved to 1-3 places (coded as 0), 4 or more places (coded as 1). Program exposure of FSWs included information about their contacts with outreach workers from government, Avahan funded programs, and/or non-governmental organisations (NGOs) in the current place. Those indicating no contacts with outreach workers were coded as "0, no exposure" and those indicating contact were coded as "1, exposed to the HIV prevention program". This measure was used as a controlling variable in the statistical analyses.

Violence victimization was based on items regarding the type of violence, physical or sexual, that FSWs experienced. Women were classified as victims of physical violence based on their affirmative responses to the following question: whether they had been physically beaten, thrown or pushed, physically hurt by any partner, brothel owner or police person either in the current or previous place of sex work in the past 12 months. Similarly, women were classified as victims of sexual violence based on their affirmative response to the following question: whether any sexual partner had physically forced you to have sex in the past 12 months either in the current or previous place of sex work.

Reproductive health risks were measured via items on FSWs' reproductive history such as number of pregnancies, number of abortions, and number of still births and experiences of RTI related symptoms in the past six months. Using information on the number of pregnancies, women were classified according to whether they had four or more pregnancies. The study assessed whether a woman ever had an abortion and the number of induced and spontaneous abortions she had experienced. Participants who reported at least one pregnancy loss due to either an abortion or still birth were classified having at least one pregnancy loss. Participants reporting at least one induced abortion were classified as having a forced termination of pregnancy. Participants reporting multiple induced abortions were classified as having multiple forced termination of pregnancies. To create a variable on experience of symptoms of RTIs in the last six months, information on the following two symptoms were used: experience of excessive vaginal discharge, and pain in the lower abdomen. Women reporting "yes" to at least one of these two symptoms were classified as experiencing RTI symptoms. Similarly, HIV risks were measured via items on inconsistent condom use in sex work with various clients in the last 24 months, in the last one week, experience of STI symptoms in the last six months and perceived HIV risk. Inconsistent condom use in the 24 months prior to the survey was coded as 1 if the FSW did not use a condom during at least one sexual encounter with a client in the places that she visited for sex work. Inconsistent condom use in the last one week was coded as 1 if the FSW has not used condoms in all sexual encounters with occasional and/or regular clients in the current place of sex work. FSWs were defined as having STI symptoms if they had experienced any of the following symptoms in the past six months: ulcers/sores in the genital area, swelling in the groin area, pain during intercourse, or frequent/sometimes painful urination. FSWs were also asked a question on the degree to which they perceive being at risk of HIV infection and the response was recoded into the two following categories: high/moderate risk, and low risk. All the sexual and HIV risk indicators are presented as dichotomous variables.

### Data analysis

The prevalence of violence and type of violence experienced were calculated for the total sample of FSWs. Differences in the experience of violence and type of violence by socio-demographic characteristics (age, education, marital status, type of sex work, duration of sex work) were assessed by χ^2 ^analyses, with significance for all analyses set at p < 0.05. A series of logistic regression models were constructed from the total sample to estimate odds ratio (ORs) and 95% confidence intervals (CIs) to assess the association between violence and indicators of reproductive health and HIV risks. Additionally, crude models and those adjusting for socio-demographic characteristics and program exposure were created. Similarly another series of regression models were constructed for the subsample of FSWs who experienced violence to assess the association between type of violence (experience of physical violence only, and the experience of sexual violence with or without physical violence) and reproductive health and HIV risks such as a high number of pregnancies, forced termination of pregnancies, RTI symptoms, inconsistent condom use, STI symptoms and low perceived HIV risk. The crude and adjusted regression models (adjusted for socio-occupation and demographics) were developed separately for ORs and adjusted ORs with 95% CIs. All statistical analyses were done using STATA (STATA Corp, 2003).

## Results

### Sample Characteristics

The median age of the study participants (N = 5,498) was 29.8 years (inter-quartile range [IQR] = 26-34 years, mean = 30.6 years, standard deviation [SD] = 6.8 years). More than one-third of the participants had no formal education; the mean number of years of education was 3.9 (SD = 3.5 years). Just over half (52%) were widowed, divorced or separated, and one-third were currently married. The median duration of sex work was 5 years (IQR = 3-8 years; mean = 6 years, SD = 5.9 years). More than one-third of FSWs were street-based; one-fifth were practicing sex work in brothel areas and equal proportions were practicing sex work from their own homes. The mean number of places FSWs had moved for sex work in the past two years was 4.4 (SD = 1.5).

### Prevalence of violence among mobile FSWs

Approximately one-third of FSWs (30.5%, n = 1,676) had experienced violence in the past 12 months. Physical violence was reported by 11% of the total sample and 19.5% reported sexual violence (Table 1). A higher proportion of FSWs with no formal education than those with a formal education (36% vs. 28%, adjusted OR = 1.4, 95% CI = 1.3-1.6), formerly married than currently married (34% vs. 27%, adjusted OR = 1.3, 95% CI = 1.1-1.5), and those who had been in sex work for more than 5 years than those in sex work for fewer years (35% vs. 28%, adjusted OR = 1.3, 95% CI = 1.2-1.5) reported experience of violence. Among FSWs who had experienced violence, sexual violence was significantly higher among women of younger age than among older women (70% vs. 56%, adjusted OR = 1.4, 95% CI = 1.1-1.8), those unmarried than those currently married (78% vs. 61%, adjusted OR = 1.6, 95% CI = 1.1-2.2), those who had been in sex work for less than 5 years than those in sex work for longer (68% vs. 57%, adjusted OR = 0.8, 95% CI = 0.6-0.9).

**Table 1:** Association between background characteristics of mobile female sex workers and experience of violence in four southern states of India (N = 5,498)

Background characteristics	All FSWs						FSWs who experienced violence				
	
	Total Sample	Percent	Experienced violence			**Adjusted OR**^**2,a **^**(95% CI)**	Type of violence experienced (%)				**Adjusted OR**^**2,b **^**(95% CI) **
						
			No (%)	Yes (%)	**p-value**^**1**^		Sub-Sample	Physical violence (%)	Sexual violence (%)	**p-value**^**1**^	
**Age**											
<30 years	2,815	51.2	70.6	29.4	.047	1.1 (1.0-1.2)*	829	29.6	70.4	<0.001	1.4 (1.1-1.8)**
≤30 years	2,683	48.8	68.4	31.6		Referent	847	43.6	56.4		Referent
**Education**											
No formal education	1,893	34.4	63.8	36.2	<0.001	1.4 (1.3-1.6)***	685	33.4	66.6	.014	1.3 (1.1-1.6)*
Formal education	3,603	65.6	72.5	27.5		Referent	990	38.8	61.2		Referent
**Marital status**					<0.001						
Currently married	1,848	33.6	72.7	27.3		Referent	505	38.8	61.2	<0.001	Referent
Unmarried	795	14.5	74.6	25.4		0.9 (0.7-1.2)	202	21.8	78.2		1.6 (1.1-2.2)**
Formerly married	2,853	51.9	66.1	33.9		1.3 (1.1-1.5)***	968	38.6	61.4		0.9 (0.7-1.1)
**Duration of sex work**					<0.001						
<5 years	3,560	64.8	71.9	28.1		Referent	1,001	32.3	67.7	<0.001	Referent
≥5 years	1,938	35.2	65.2	34.8		1.3 (1.2-1.5)***	675	43.1	56.9		0.8 (0.6-0.9)*
**Type of sex work^c^**					<0.001						
Brothel	1,182	21.5	72.9	27.1		Referent	320	31.9	68.1	0.002	Referent
Lodge	437	7.9	69.6	30.4		1.3 (1.0-1.7)*	133	27.1	72.9		1.3 (0.8-2.2)
Street	1,998	36.3	68.7	31.3		1.3 (1.1-1.6)**	625	40.2	59.8		0.8 (0.6-1.0)
Home	1,095	19.9	72.1	27.9		1.0 (0.9-1.3)	306	41.8	58.2		0.8 (0.5-1.0)
Highway	786	14.3	62.8	37.2		1.7 (1.4-2.1)***	292	33.2	66.8		0.9 (0.6-1.2)
**Degree of mobility**											
1-3 places	1,687	30.7	68.8	31.2	0.24	Referent	526	34.0	66.0	0.13	Referent
4+places	3,811	69.3	69.8	30.2		0.9 (0.8-1.0)	1,150	37.8	62.2		0.9 (0.7-1.1)
**Total**	**5,498**	**100.0**	**69.5**	**30.5**			**1,676**	**36.6**	**63.4**		

^1 ^Chi-sq. test; ^2 ^AOR - Adjusted Odds Ratio (adjusted for all the variables in this table)

* p < 0.05; ** p < 0.01; *** p < 0.001

^a ^Dependent variable: Experienced violence (No - 0, Yes - 1).

^b ^Dependent variable: Type of violence (Physical violence - 0, Sexual violence - 1)

^c ^Type of sex work: As recorded by the interviewer based on recruitment site.

### Association between experience of violence and reproductive health and HIV risks

Results indicate that FSWs who had experienced any violence (physical or sexual) were significantly more likely to be vulnerable to both reproductive health and HIV risks (Table 2). For example, as compared to FSWs who did not experience violence, FSWs who experienced any violence had a higher number of pregnancies (adjusted OR = 1.2, 95% CI = 1.0-1.6), had experienced pregnancy loss (31.1% vs. 24.5%; adjusted OR = 1.4, 95% CI = 1.2-1.6), had experienced forced termination of pregnancy (adjusted OR = 2.4, 95% CI = 2.0-2.7), and had multiple forced termination of pregnancies (adjusted OR = 2.2, 95% CI = 1.7-2.8), which are factors indicative of vulnerability to reproductive health risks. Similarly, women reporting the experience of violence were significantly more likely than those who did not experience violence to report inconsistent condom use in the past two years (adjusted OR = 2.0, 95% CI = 1.7-2.3), current inconsistent condom use (adjusted OR = 1.7, 95% CI = 1.4-2.0), symptoms of STI in the last six months (adjusted OR = 2.1, 95% CI = 1.8-2.4) and a high perception of HIV risk (adjusted OR = 1.8, 95% CI = 1.6-2.0), which are factors indicative of HIV risk.

**Table 2:** Results of the logistic regression analyses of mobile FSWs, with or without the experience of violence, and reproductive health risk and HIV risk (N = 5498)

Dependent Variable	Among FSWs who did not experience violence	Among FSWs who experienced violence	**OR**^**1,a,b **^**(95% CI)**	**Adjusted OR**^**2,a,b **^**(95%CI)**
**Reproductive health risks**				

FSWs who reported:				
**≥4 pregnancies**				
Yes	13.5	16.5	1.3(1.1-1.4)**	1.2(1.0-1.6)*
No	86.5	83.5	Referent	Referent
**Any pregnancy loss**				
Yes	24.5	31.1	1.3(1.2-1.5)***	1.4(1.2-1.6)***
No	75.5	68.9	Referent	Referent
**Any forced termination of pregnancy**				
Yes	11.8	23.5	2.3(1.9-2.6)***	2.4(2.0-2.7)***
No	88.2	76.5	Referent	Referent
**Multiple forced termination of pregnancies**				
Yes	4.0	7.9	2.0(1.6-2.6)***	2.2(1.7-2.8)***
No	96.0	92.1	Referent	Referent
**Symptoms of RTI^3 ^(in last six months)**				
Yes	54.9	64.5	1.5(1.3-1.6)***	1.5(1.3-1.7)***
No	45.1	35.5	Referent	Referent

**HIV risks**				

**Inconsistent condom use (in last two years)**				
Yes	47.8	63.1	1.8(1.6-2.1)***	2.0(1.7-2.3)***
No	52.2	36.9	Referent	Referent
**Inconsistent condom use (in last 7 days)**				
Yes	41.4	52.1	1.5(1.4-1.7)***	1.7(1.4-2.0)***
No	58.6	47.9	Referent	Referent
**STI^4 ^symptoms (in last six months)**				
Yes	51.0	68.4	2.0(1.8-2.3)***	2.1(1.8-2.4)***
No	49.0	31.6	Referent	Referent
**Perception of HIV risk**				
High/Moderate	34.8	49.6	1.8(1.6-2.1)***	1.8(1.6-2.0)***
Low	65.2	50.1	Referent	Referent

* p < 0.05 ** p < 0.01 *** p < 0.001

^1 ^Odds ratio (unadjusted)

^2 ^Adjusted odds ratio: adjusted for age, education, marital status, duration of sex work, type of sex work, program exposure, mobility

^3 ^Women reporting experience of any of the following two symptoms in the six months prior to the survey: excessive vaginal discharge, or pain in lower abdomen

^4 ^Women reporting experience of any of the following four symptoms in the six months prior to the survey: ulcers/sores in genital area, swelling in groin area, pain during intercourse, or frequent/sometimes painful urination

^a ^Dependent variable: Predicting 'yes' on each of the dependent variables for reproductive health and HIV risks

^b ^Odds ratios are presented for key independent variable: Experienced no violence = Reference Category; Experienced violence = 1).

### Association between experience of sexual and physical violence and reproductive health and HIV risks

Analyses were further conducted to understand the effect of different types of violence on reproductive health and HIV risks (Table 3). FSWs who experienced sexual violence with/without physical violence were significantly more likely than those who experienced only physical violence to report inconsistent condom use in the last two years (adjusted OR = 1.8, 95% CI = 1.4-2.3), current inconsistent condom use (adjusted OR = 1.8, 95% CI = 1.5-2.3), and symptoms of STI in the last six months (adjusted OR = 1.3, 95% CI = 1.1-1.7). The multivariate models documented no significant association between type of violence and reproductive health risks except for symptoms of RTI, indicating that abuse in general is an important determinant of reproductive health risk.

**Table 3:** Results of the logistic regression analyses of mobile FSWs experience of physical violence or sexual violence (among those who experienced violence) and reproductive health and HIV risks (n = 1,676)

Dependent variables	Experienced physical violence	Experienced sexual violence (with/without physical violence)	**OR**^**1,a **^**(95% CI)**	**Adjusted OR**^**2,a **^**(95%CI)**
**Reproductive health risks**				

FSWs who reported:				
**≤4 pregnancies**				
Yes	18.9	15.1	0.8 (0.6-0.9)*	0.9 (0.7-1.3)
No	81.1	84.9	Referent	Referent
**Any pregnancy loss**				
Yes	33.1	29.9	0.9 (0.7-1.0)	0.9 (0.7-1.1)
No	66.9	70.1	Referent	Referent
**Any forced termination of pregnancy**				
Yes	24.3	23.1	0.9 (0.7-1.1)	0.9 (0.7-1.1)
No	75.7	76.9	Referent	Referent
**Multiple forced termination of pregnancies**				
Yes	6.8	8.5	1.2 (0.9-1.8)	1.1 (0.8-1.7)
No	93.2	91.5	Referent	Referent
**Symptoms of RTI^3 ^(in last six months)**				
Yes	61.1	66.5	1.2 (1.0-1.5)**	1.3 (1.0-1.7)**
No	38.9	33.5	Referent	Referent

**HIV risks**				

**Inconsistent condom use (in last two years)**				
Yes	54.1	68.4	1.8 (1.5-2.2)***	1.8 (1.4-2.3)***
No	45.9	31.6	Referent	Referent
**Inconsistent condom use (in last 7 days)**				
Yes	41.5	58.2	1.9 (1.6-2.4)***	1.8 (1.5-2.3)***
No	58.5	41.8	Referent	Referent
**STI^4 ^symptoms (in last six months)**				
Yes	65.5	70.1	1.2 (1.0-1.5)*	1.3 (1.1-1.7)**
No	34.5	29.9	Referent	Referent
**Perception of HIV risk**				
High/moderate	53.4	47.9	0.8 (0.6-0.9)**	0.9 (0.8-1.1)
Low	46.6	52.1	Referent	Referent

* p < 0.05 ** p < 0.01 *** p < 0.001

^1 ^Odds ratio (unadjusted)

^2 ^Adjusted odds ratio: adjusted for age, education, marital status, duration of sex work, type of sex work, program exposure, mobility

^3 ^Women reporting experience of any of the following two symptoms in the six months prior to the survey: excessive vaginal discharge, or pain in lower abdomen

^4 ^Women reporting experience of any of the following four symptoms in the six months prior to the survey: ulcers/sores in genital area, swelling in groin area, pain during intercourse, or frequent/sometimes painful urination

^a ^Dependent variable: Predicting 'yes' on each of the dependent variables for reproductive health and HIV risks

^b ^Odds ratios are presented for key independent variable: Experienced physical violence only = Reference Category; Experienced sexual violence with or without physical violence = 1.

## Discussion

Findings from this study indicate that nearly one-third of mobile FSWs in high HIV prevalence states of India experience physical or sexual violence. The experience of violence is associated with increased pregnancies, pregnancy loss, RTI symptoms, inconsistent condom use, STIs symptoms and high perception of HIV risk. Violence victimization in general among FSWs is associated with both reproductive health and HIV risks. However, FSWs who had experienced sexual violence were more likely to report HIV risks than those who experienced only physical violence. These findings indicate that reproductive health risks continue to be a concern among generally abused FSWs and suggest the need for interventions to address the reproductive health needs of FSWs along with existing HIV risk reduction programs.

The reported prevalence of violence among mobile FSWs in this study is consistent with previous research among all FSWs [11,15] and among women in the general population [7,19], suggesting that this group of FSWs face a double jeopardy because in addition to being vulnerable to experiencing violence they are also socially stigmatized. As many as one-third of FSWs experience violence; this experience of violence is higher among FSWs who have no formal education, formerly married and those in sex work for more than five years.

Among violence episodes, the most critical - sexual violence-- is experienced to a larger extent than physical violence; this is also true of sex workers in most countries in the Asian region [9,23,25]. These results suggest that neither extensive HIV prevention programmatic attention [30,31] nor the National AIDS Control Policy [32] have been adequate to reduce violence, particularly to protect FSWs who are the most disadvantaged.

This study is amongst the first to examine the relationship between the experience of violence and reproductive health and HIV risks among mobile FSWs in India, and advances prior work from multiple settings demonstrating that violence victimization is linked to HIV risk behaviors in India and elsewhere [15,33]. Additionally, the high level of reproductive health risks identified to be associated with violence indicate the need to develop new strategies in intervention programs that will address the adverse reproductive health circumstances of the abused FSWs and the violence. Similarly, the higher levels of inconsistent condom use in sex with clients and symptoms of STI among abused FSWs as compared to FSWs who have not experienced violence points to increased HIV risk among FSWs who face violence. These results are consistent with previous work in other developing countries, which indicate that the threat of physical or sexual violence results in sex workers 'agreeing to have sex without condoms' thus potentially exposing them to HIV infection [34]. Evidence from studies of women in the general population in South Asia too have documented the high rates of intimate partner violence [3,7] and its impact on the risk of HIV infection.

Prior to this study, the understanding of the effects of violence victimization on FSWs' reproductive health in India was limited. Similar to abused women in the general population, abused FSWs also continue to experience reproductive health and HIV risks and the support systems of FSWs are more limited than those of women in the general population because of the clandestine nature of sex work in India [35].

Although findings from this study offer important insights into violence victimization among FSWs in India and its association with reproductive health and HIV risks, they must be interpreted in the light of certain study limitations. First, responses to violence victimization, STIs, condom use and reproductive health outcomes are based on self-reports and are therefore vulnerable to significant social desirability and recall biases, and under reporting. The use of experienced research staff linked with training and local support was designed to increase respondents' comfort and reduce social desirability; moreover, the use of short time-frames for recall was designed to reduce recall biases. However, the self-reported symptoms of STI may be an underestimate given that many FSWs with STIs may not experience any symptoms, and some of those reporting symptoms may not actually have an STI [36]. Second, analyses are cross-sectional; thus, causality cannot be assumed in linkages between indicators of reproductive health and violence victimization. Finally, findings are specific to mobile FSWs from four high prevalence states of India, and cannot be generalized to other FSWs within India. Nonetheless, current findings are consistent with those observed in previous cross-national studies of violence victimization among FSWs [11,37], and advances the knowledge on association between violence and reproductive health and HIV risks in this marginalized and socially stigmatized group.

The pervasiveness of violence among FSWs and its strong association with reproductive health and HIV risks indicates the crucial need for violence prevention interventions among FSWs in India. Findings from this study are timely because the up-scaling of the community mobilization strategy in HIV prevention interventions is underway; this could provide a window of opportunity for these programs to intervene in areas where violence reported to be high as well as to integrate reproductive health services for FSWs within HIV risk reduction programs. Interventions to promote safe sex among FSWs must be part of an overall effort to ensure their safety from contextual risk in terms of sex work vulnerability towards violence and abuse. Empowering and equipping FSWs with self-defense skills to address violence could be another effective strategy. Other strategies to address violence among FSWs could include helping FSWs to identify risky clients, build awareness about their legal rights, assist them to network with collectives, and inform them about how to prevent abuse. Efforts should also focus on FSWs' reproductive health needs, and should integrate family planning education, particularly in sexual relations with non-paying partners or husbands. While major efforts are under way to reduce the risk of FSWs acquiring and transmitting HIV, it is vital that existing interventions are expanded to integrate reproductive health services for FSWs and address violence within such contexts.

## Conclusion

The data presented here highlight the persistent high rates of violence among mobile FSWs and their association with adverse reproductive health outcomes in addition to HIV risks. These results call for strengthened initiatives using a multi-faceted approach to address violence and reproductive health issues in HIV prevention programmes for FSWs. An ongoing and future initiative to address individual, community and societal level structural factors to create an enabling environment is important to improved reproductive and sexual health for under-served populations such as FSWs.

## Competing interests

The authors declare that they have no competing interests.

## Authors' contributions

SNS led conceptualization, conducted all analyses, and led manuscript development for this paper. NS assisted with the conceptualization, provided input into analytic approach, and assisted with significant writing and editing of the paper. MB assisted with the analyses and writing of the paper. RKV assisted with interpretation of study findings. AKJ assisted with conceptualization of analytic approach, and interpretation of study findings. All authors participated as described above and both saw and approved this final submitted manuscript.

## Pre-publication history

The pre-publication history for this paper can be accessed here:

http://www.biomedcentral.com/1471-2458/11/357/prepub
